# Right ventricular to pulmonary artery uncoupling is an early predictor of poor outcome in wild-type transthyretin amyloid cardiomyopathy

**DOI:** 10.1007/s10554-025-03394-x

**Published:** 2025-04-10

**Authors:** Giulio Sinigiani, Laura De Michieli, Matteo d’Addazio, Lisa Portalone, Monica De Gaspari, Alessandro Lupi, Alessandro Zorzi, Francesco Tona, Cristina Basso, Martina Perazzolo Marra, Sabino Iliceto, Domenico Corrado, Stefano Nistri, Donato Mele, Alberto Cipriani

**Affiliations:** 1 Department of Cardio-Thoraco-Vascular Sciences and Public Health, University of Padua, Padua, Italy; 2 Cardiology Unit, University Hospital of Padua, Via N. Giustiniani 2, 35121 Padua, Italy; 3 Cardiovascular Pathology Unit, University Hospital of Padua, Via A. Gabelli 61, 35121 Padua, Italy; 4 Department of Cardiac, Thoracic, Vascular Sciences and Public Health, University of Padua, Via N. Giustiniani 2, 35121 Padova, Italy

**Keywords:** Transthyretin cardiac amyloidosis, Echocardiography, Right ventricular strain, Right ventricle to pulmonary artery coupling, Prognosis

## Abstract

Non-invasive right ventricular to pulmonary artery (RV-PA) uncoupling assessment has prognostic value in patients with heart failure (HF). Little is known about its application in patients with wild-type transthyretin amyloid cardiomyopathy (wtATTR-CM). This single-centre retrospective study included consecutive patients with wtATTR-CM diagnosis undergoing 2D echocardiogram. RV-PA uncoupling was evaluated with the ratios between tricuspid annular plane systolic excursion (TAPSE), RV free wall longitudinal strain (RVFWLS) or RV four-chamber longitudinal strain (RV4CLS) and pulmonary artery systolic pressure (sPAP). Primary endpoint was the composite of all-cause mortality and HF hospitalisation. Overall, 100 patients (91% males, median age 81 years, 85% in National Amyloid Centre (NAC) stage *≤* 2, 18% in NAC stage Ia and 82% in New York Heart Association class *≤* II) were enrolled. Over a 16-months follow up (Q1-Q3:12–24), the primary endpoint occurred in 37 patients (37%). TAPSE/sPAP (HR 0.04, 95% CI 0.01–0.24, *p* < 0.001), RVFWLS/sPAP (HR 0.07, 95% CI 0.01–0.41, *p* = 0.003) and RV4CLS/sPAP (HR 0.06, 95% CI 0.01–0.53, *p* = 0.011) emerged as independent predictors of the primary endpoint and showed incremental risk prediction compared with TAPSE, RVFWLS and RV4CLS, considered as separate parameters. No differences in outcome risk prediction were observed among TAPSE/sPAP, RVFWLS/sPAP and RV4CLS/sPAP (*p* > 0.05). RV-PA uncoupling, as assessed by different echocardiography modalities, is an early predictor of poor outcome in patients with wtATTR-CM.

**Graphical Abstract:**

**Figure:**
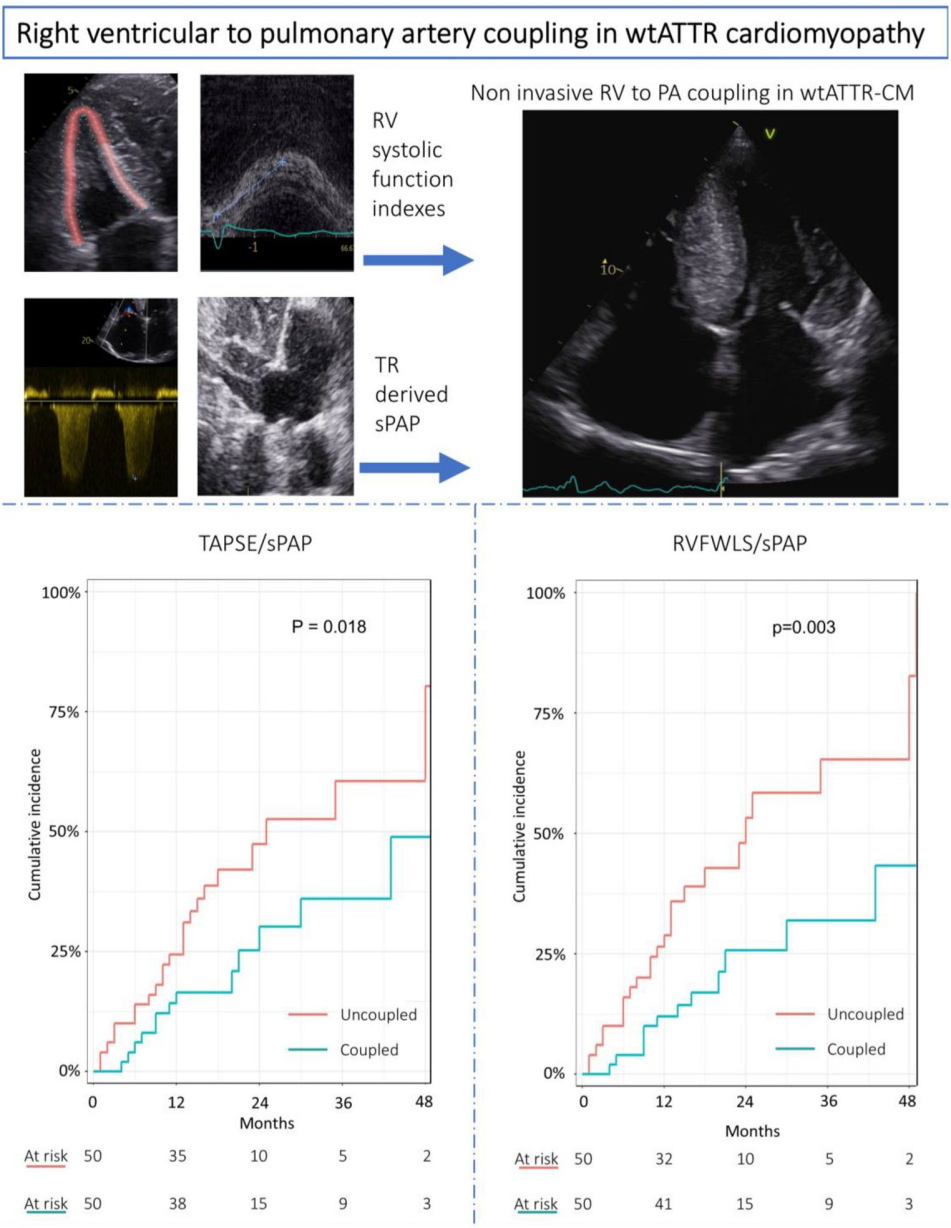
Central illustration. Prognostic value of non-invasive right to pulmonary artery coupling in wild-type transthyretin cardiomyopathy. Bad outcome is observed in patients with worse RV-PA coupling ratios. Abbreviations: PA = pulmonary artery; RV = right ventricle; RVFWLS = right ventricular free-wall longitudinal strain; sPAP = systolic pulmonary artery pressure; TAPSE = tricuspid annulus plane systolic excursion; TR = tricuspid regurgitation; wtATTR-CM = wild-type transthyretin cardiomyopathy

**Supplementary Information:**

The online version contains supplementary material available at 10.1007/s10554-025-03394-x.

## Introduction

Wild-type transthyretin amyloid cardiomyopathy (wtATTR-CM) is a sporadic non-inherited cardiac disease characterized by the deposition of misfolded transthyretin in the heart, causing a progressive disruption of cardiac structure and function [1–3]. This condition is typically characterized by increased left ventricular (LV) wall thickness and stiffness, predisposing to impaired diastolic function and heart failure with preserved ejection fraction (HFpEF) [2]. A significant epidemiological increase of this condition has been observed in recent times, due to the adoption of non-invasive diagnostic algorithms [4], and patients are currently more frequently early diagnosed, showing no or mild symptoms, lower disease stage, and more favourable structural abnormalities at diagnosis [5]. At the same time, novel disease-modifying agents have been identified to stop or delay the progression of wtATTR-CM [6], so that there is an urgent clinical need to identify early predictors of poor outcome that could prompt the initiation of ATTR-targeted therapy, also in asymptomatic or early stages patients.

The right ventricle (RV) to pulmonary artery (PA) coupling is defined as the ratio of RV function to pulmonary vascular afterload and is traditionally assessed by means of echocardiography using the ratio between tricuspid annular plane systolic excursion (TAPSE) and systolic pulmonary artery pressure (sPAP) [7–9]. Recently, other ratios using RV strain function indexes have been proposed and validated [10–13]. The RV-PA uncoupling has emerged as a strong prognostic factor in patients with HF [14–20] and also in historical mixed cohorts of light-chain and ATTR cardiomyopathy patients [7, 8]. However, little evidence is available about its pathophysiological and clinical meaning in wtATTR– CM only, particularly in contemporary cohorts, including early diagnosed and less sick patients [5].

## Methods

### Study design and study population

The Cardiac Amyloidosis Outpatient Clinic of University Hospital of Padua (Italy) is a tertiary centre for evaluation of all patients with established or suspected amyloid cardiomyopathy. At the time of the first visit, all patients undergo a routine clinical evaluation, including family and personal history, physical examination, biomarkers analysis, resting 12-lead ECG, and 2-dimensional transthoracic echocardiography. This is a single-centre observational longitudinal study, enrolling a consecutive series of patients with a definitive diagnosis of wtATTR-CM, established according to the *Gillmore algorithm* [4], between January 2018 and January 2023. All first echocardiographic exams of these patients performed in our Institution were retrieved and re-analysed focusing on RV-PA uncoupling assessment. Exclusion criteria were inadequate image quality for strain analysis (frame rate < 50 frames per seconds or inability to accurately visualize the RV from base to apex or to perform adequate speckle - tracking analysis on any RV segments), insufficient data for a reliable assessment of systolic pulmonary artery pressure, and history of severe chronic obstructive pulmonary disease, severe obstructive sleep apnoea syndrome or pulmonary embolism. All patients underwent genetic testing, and those diagnosed with hereditary ATTR-CM were not included in this study, due to the low prevalence in our region [21], the highly variable phenotypes [22] and the different clinical and echocardiographic features compared with wtATTR-CM [23]. Patients were systematically followed up from the date of our first cardiologic evaluation (baseline), to avoid any time referral bias. The clinical data recorded within ± 1 months from the baseline included all the following: (I) medical history and physical examination, (II) ECG and (III) laboratory exams. The local regional Institutional Review Board approved the study, and the investigators obtained local institutional review board approvals for the retrospective collection of anonymous data. The study was conducted according to the Declaration of Helsinki, and informed consent was obtained according to the local review board policies.

### Clinical history, electrocardiography and biomarkers

Careful clinical history, ongoing medical therapy, and data regarding New York Heart Association (NYHA) class and National Amyloidosis Centre (NAC) stage [24] at baseline, including NAC Ia stage [25], were collected. Tafamidis in Italy was approved in October 2021, and the Italian Medicines Agency authorized its reimbursement exclusively in patients with ATTRwt-CM and NYHA class I or II [26]. Disease modifying therapy at baseline or during the follow up was noted. Further details are provided in Supplemental Materials.

### Echocardiography

Echocardiographic images were acquired using a Vivid 9 ultrasound system (General Electric Medical System, Milwaukee, USA), and analysis was independently carried out in post– processing by a trained cardiologist blinded to patients’ history using the EchoPAC software v.204 (General Electric Medical System, Milwaukee, USA). American Society of Echocardiography and the European Association of Cardiovascular Imaging recommendations [27, 28] were careful followed. TAPSE was measured using M-mode echocardiography at tricuspid annulus level. Longitudinal strain (LS) was quantified using a region of interest including both right ventricle free wall (RVFW), with adequate width to cover its thickness, and interventricular septum (IVS). RVFW longitudinal strain (RVFWLS) was measured as the average of the strain values of the three segments of the RVFW; RV four-chamber LS (RV4CLS) was measured as the average of the strain values of the six segments of the RVFW and IVS [29]. Right atrium longitudinal strain (RALS) was calculated as established in literature [28], limited to reservoir phase due to high prevalence of atrial fibrillation in our cohort. Systolic pulmonary artery pressure (sPAP) was calculated using the formula: 4*(peak velocity of TR)^2^ + estimated right atrial pressure. The latter was derived on the inferior vena cava diameter and collapsibility [30]. RV-PA uncoupling parameters (i.e., RVFWLS/sPAP and RV4CLS/sPAP) were positivized for easier comprehension. Further details are provided in Supplemental Materials.

### Outcomes and statistical analysis

Continuous baseline characteristics were expressed as median with 25th and 75th percentiles [Q1– Q3] and were compared using the Mann– Whitney test. Categorical variables were expressed as absolute numbers and percentages and were compared using the chi-square (χ2) test. The primary endpoint was the composite of all-cause death and HF hospitalisation. The latter was defined as an admission to hospital for HF symptoms and need for intravenous diuretic therapy. Survival analysis was performed with a Cox proportional hazards regression, with univariable and multivariable models. The number of variables entered into the multivariable model was limited according to the number of events, based on the principle of not having more than one variable every 10 events. Thus, multiple models were built to test the predictive value of RV systolic function parameters and RV-PA uncoupling values, adjusting for covariates that were both statistically significant at univariate analysis (*p* < 0.05) and selected on the basis of their clinical relevance coupled with absence of collinearity. Candidate predictors included HF presentation, defined as HF hospitalization requiring intravenous diuretic therapy before the diagnosis, N-terminal pro-brain natriuretic peptide (NT-proBNP) and furosemide intake > 50 mg [24, 31, 32]. To correctly assess the impact of disease modifying therapy, a dedicated time– dependent Cox’s regression analysis was carried out. Overfitting was eventually tested with a 10-fold cross validation of each model, by comparison of original and cross-validated C-index with a threshold in difference of 0.5. For independent predictors, median values were used to draw the primary endpoint cumulative incidence curves using the Kaplan Meier method and the log-rank test. For estimating the incremental prognostic value of the RV-PA uncoupling indexes over the uncoupled RV systolic function parameters, the time-dependent areas under the curve (AUC) of the corresponding ROCs of each were evaluated and compared as previously defined [33]. All tests were 2-tailed, and a *p* < 0.05 was considered statistically significant. Statistical analyses were conducted using the SPSS software version 26.0 statistical package and the RStudio software version 4.3.1.

## Results

### Study population

**Fig. 1:**
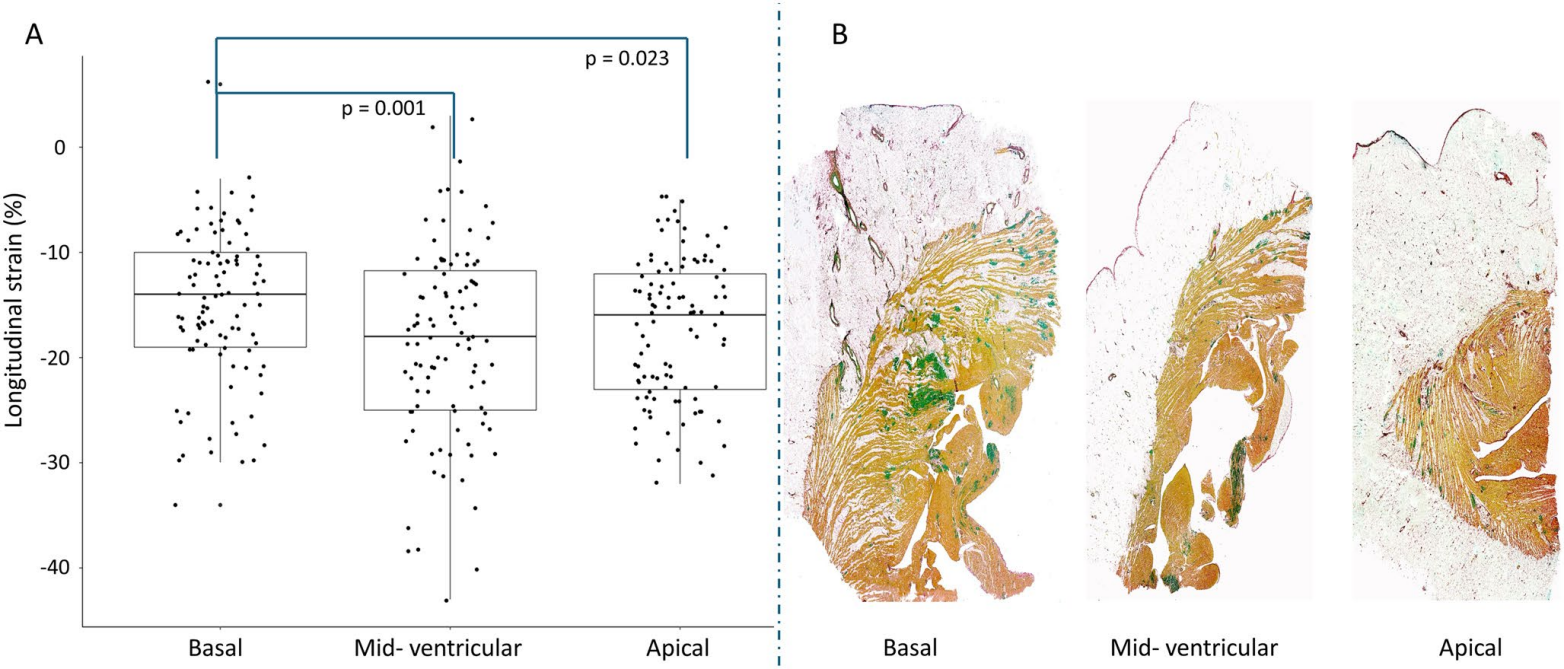
Right ventricular (RV) free wall longitudinal strain and amyloid infiltration. Panel (**A**) Longitudinal strain values calculated in the basal segments of the RV free wall are significantly lower than mid-ventricular and apical ones. Panel (**B**) Histological panoramic view of the RV free wall of a 81-year-old female patient with wtATTR-CM, showing larger amyloid infiltration (sulphated alcian blue stain) in the basal segments, compared with the mid-ventricular and apical ones

Among 202 patients diagnosed with cardiac amyloidosis between January 2018 and January 2023, 135 (68%) had a diagnosis of wtATTR-CM. After exclusion of those patients with inadequate image quality (n = 35), 100 (74%) constituted the study population (Supplemental Fig. 1). Baseline characteristics are shown in Table 1. Most patients were male (n = 91, 91%) with a median age of 81 (75–85) years, a NYHA class I or II (n = 82, 82%) and a NAC stage I or II (n = 85, 85%). The majority was treated with disease modifying therapy (n = 53, 53%), with a median time from diagnosis of 3 (1–8) months, and a median follow up in therapy of 15 (10–18) months (Supplemental Table 1). Considering echocardiogram data, median values of E/A and E/e’ ratios were 2.1 (1.1 to 2.7) and 16.1 (13.4–19.4). Median values of RVFWLS and RV4CLS were − 16.5% and − 12.1%. On segmental analysis of the RVFW, LS were − 14% (-19 to -10), -18% (-25 to -11) and − 16% (-23 to -12) for basal, mid, and apical segments, respectively (Fig. 1, panel A). Regarding RV-PA coupling non-invasive parameters, median values of TAPSE/sPAP, RVFWLS/sPAP and RV4CLS/sPAP were 0.45 mm/mmHg, 0.46%/mmHg and 0.33%/mmHg, respectively. Intra- and inter-readers intraclass correlation coefficients (ICC) are provided in Supplemental Table 2. A moderate correlation (Spearman’s R -0.37, -0.36 and − 0.37) was found between E/e’ ratio and TAPSE/sPAP, RVFWLS/sPAP and RV4CLS/sPAP, respectively (Supplemental Fig. 3).

**Table 1:** Population characteristics stratified according to composite endpoint occurrence

Variable	Total population *N* = 100	No Endpoint *N* = 63	Endpoint *N* = 37	*p*
**Clinical characteristics**				
Age (years)	81 (75–85)	80 (75–84)	81 (76–85)	0.4
Male sex (%)	91 (91)	56 (89)	35 (95)	0.5
NYHA class > II (%)	18 (18)	10 (16)	8 (22)	0.6
HF presentation (%)	52 (54)	27 (44)	25 (69)	**0.020**
COPD (%)	9 (9)	8 (15)	1 (3)	0.3
OSAS (%)	1 (1)	1 (2)	0 (0)	0.9
NAC stage I (%)	63 (63)	44 (70)	19 (51)	0.06
NAC stage II (%)	22 (22)	11 (17)	11 (30)	0.1
NAC stage III (%)	15 (15)	8 (15)	7 (19)	0.8
**Medical therapy**				
Furosemide (%)	69 (69)	38 (60)	31 (84)	**0.015**
Dose of furosemide (mg)	25 (0–50)	25 (0–50)	50 (25–100)	**0.006**
β–blockers (%)	53 (53)	36 (57)	17 (46)	0.3
ACE-i/ARBs/ARNI (%)	50 (50)	31 (49)	19 (51)	1
SGLT2i (%)	5 (5)	3 (8)	2 (6)	0.9
MRA (%)	39 (39)	21 (33)	18 (49)	0.1
Disease modifying therapy (%)	53 (53)	41 (65)	12 (32)	**0.002**
Time of DMT starting from diagnosis (months)	3 (1–8)	3 (1–8)	6 (1–11)	0.4
**Electrocardiogram characteristics**				
AF (%)	49 (49)	30 (48)	19 (51)	0.8
LQRSV (%)	31 (31)	23 (38)	8 (22)	0.1
QRS duration (ms)	118 (101–144)	111 (99–143)	135 (117–147)	**0.007**
**Biochemical characteristics**				
NTproBNP (ng/L)	1777 (815–4896)	1610 (745–4221)	1921 (928–6010)	0.1
eGFR (ml/min/m2)	60 (50–77)	62 (53–77)	57 (45–75)	0.3
Hs-TnI (ng/L)	78 (41–138)	60 (36–121)	92 (54–234)	**0.020**
**Echocardiogram characteristics**				
IVS (mm)	18 (16–20)	18 (15–20)	18 (16–20)	0.9
PW (mm)	15 (14–17)	16 (14–18)	15 (14–17)	0.6
RWT	0.72 (0.59–0.83)	0.74 (0.57–0.86)	0.70 (0.59–0.80)	0.3
LV mass (gr)	307 (258–387)	284 (250–387)	314 (269–388)	0.4
LV EDVi (ml/m2)	56 (46–67)	54 (47–64)	60 (43–72)	0.3
LV EF (%)	52 (44–57)	53 (43–58)	51 (44–56)	0.4
LV SVi (ml/m2)	27.2 (22.2–33.1)	29 (23–35)	27 (22–33)	0.9
LV GLS (-%)	11 (8–13)	11 (8–13)	11 (7–12)	0.6
E/A	2.1 (1.1–2.7)	1.5 (0.9–2.7)	2.6 (2.1–2.8)	**0.028**
E/e’	16.1 (13.4–19.4)	15.8 (11.9–19.4)	16.7 (14.4–19.2)	0.1
E/e’>14 (%)	68 (69)	37 (60)	31 (86)	**0.007**
Restrictive filling pattern (%)	28 (28)	13 (21)	15 (41)	**0.032**
LAVi (ml/m2)	52.5 (42.3–65.4)	49.7 (41.9–60.5)	59.4 (47.0–70.9)	**0.031**
RAVi (ml/m2)	45.5 (34.4–57.1)	43.4 (33.6–56.1)	50.2 (38.7–63.2)	0.06
RALS (%)	13.1 (8.1–15.9)	15.1 (8.9–16.1)	10.0 (5.3–13.8)	**0.034**
RV thickness (mm)	7.0 (4.2–9.0)	7 (4–8)	7 (5–9)	0.3
RV EDAi (cm2/m2)	11.6 (9.8–12.9)	11.4 (9.6–12.5)	12.4 (10.6–14.3)	**0.008**
TAPSE (mm)	16.5 (13.0–20.0)	17.1 (14.0–20.4)	15.1 (12.1–19.2)	**0.038**
RV FAC (%)	35.5 (30.0–41.8)	36 (30–42)	33 (31–42)	0.8
RVFWLS (-%)	16.5 (12–21.5)	17.9 (13–22)	15.0 (11–20)	**0.043**
RV4CLS (-%)	12.1 (9.1–16.6)	12.5 (9.2–16.8)	11 (8–15.3)	0.2
sPAP (mmHg)	35 (26–45)	30 (22–42)	39 (33–47)	**0.003**
TAPSE/sPAP (mm/mmHg)	0.45 (0.33–0.72)	0.50 (0.36–0.83)	0.38 (0.27–0.52)	**0.001**
RVFWLS/sPAP (%/mmHg)	0.46 (0.31–0.72)	0.49 (0.35–0.87)	0.39 (0.27–0.52)	**0.001**
RV4CLS/sPAP (%/mmHg)	0.33 (0.23–0.52)	0.39 (0.26–0.62)	0.28 (0.20–0.42)	**0.006**
Trivial TR (%)	34 (34)	29 (46)	5 (14)	**0.001**
Mild TR (%)	45 (45)	22 (35)	23 (62)	**0.010**
Moderate TR (%)	20 (20)	12 (19)	8 (22)	0.8
Severe TR (%)	1 (1)	0 (0)	1 (3)	0.4
Severe MR (%)	0 (0)	0 (0)	0 (0)	1
Severe AS (%)	1 (1)	0 (0)	1 (3)	0.4
Severe pericardial effusion (%)	1 (1)	1 (2)	0 (0)	1
Pleural effusion (%)	8 (8)	4 (6)	4 (11)	0.5

Quantitative variables expressed as median value (25th − 75th percentile). Qualitative variables expressed as absolute number (%). Abbreviations: ACE-i = Angiotensin converter enzyme inhibitor; AF = atrial fibrillation; ARBs = angiotensin receptor blockers; ARNI = angiotensin receptor neprilysin receptor inhibitors; AS = aortic stenosis; COPD = chronic obstructive pulmonary disease; DMT = disease modifying therapy; EDAi = end diastolic area indexed; EDVi = end diastolic volume indexed; EF = ejection fraction; eGFR = estimated glomerular filtration rate; FAC = fractional area change; GLS = global longitudinal strain; HF = heart failure; Hs–TnI = high sensitivity troponin I; IVS = interventricular septum; LAVi = left atrium volume indexed; LQRSV = low QRS voltages; LV = left ventricle; MR = mitral regurgitation; MRA = mineralocorticoids receptor antagonist; NAC = National Amyloid Centre; NYHA = New York Heart Association; NTproBNP = N-Terminal pro brain natriuretic peptide; OSAS = obstructive sleep apnoea syndrome; PW = posterior wall; RALS = right atrium longitudinal strain; RAVi = right atrium volume indexed; RVFWLS = RV free wall longitudinal strain; RV4CLS = RV 4-chamber longitudinal strain; RWT = relative wall thickness; SGLT2i = Sodium glucose transporter 2 inhibitors; sPAP = systolic pulmonary artery pressure; SVi = stroke volume indexed; RV = right ventricle; TAPSE = tricuspid annulus plane systolic excursion; TR = tricuspid regurgitation

### Primary endpoint and follow-up

During a median follow-up time of 16 months (Q1-Q3: 12–24), the primary endpoint occurred in 37 (37%) patients. All-cause death and heart failure hospitalization occurred in 22 (22%) and 25 (25%) patients, respectively. Compared with those without, patients with primary endpoint had a significantly higher prevalence of HF presentation (69% vs. 44%, *p* = 0.020), and were more frequently treated with furosemide (84% vs. 60%, *p* = 0.015) at higher dose (50 mg vs. 25 mg, *p* = 0.006), and less frequently with disease modifying therapy drugs (32% vs. 65%, *p* = 0.002) (Table 1). Considering laboratory test, patients with primary endpoint had higher high sensitivity troponin I value (92 vs. 60 ng/L, *p* = 0.020), but no significantly differences emerged for N terminal pro-brain natriuretic peptide (NT-proBNP) (1921 vs. 1610 ng/L, *p* = 0.1) or estimated glomerular filtration rate (eGFR) (57 vs. 62 ml/min/m^2^, *p* = 0.3). On echocardiography, they had more frequently a LV restrictive filling pattern (41% vs. 21%, *p* = 0.032), with higher E/A ratio (2.6 vs. 1.5, *p* = 0.028) and left atrium volume indexed (59.4 ml/m^2^ vs. 49.7 ml/m^2^, *p* = 0.031). No significant differences among groups emerged in LV ejection fraction (51% vs. 53%, *p* = 0.4) or LV global longitudinal strain (-11% vs. -11%, *p* = 0.6). Compared with those without, patients with primary endpoint had significantly lower RALS (10.0% vs. 15.1%, *p* = 0.034), significantly higher RV end-diastolic indexed area and sPAP (12.4 cm^2^/m^2^ vs. 11.4 cm^2^/m^2^, *p* = 0.008 and 39 mmHg vs. 30 mmHg, *p* = 0.003) and more impaired RV systolic function, either evaluated with TAPSE (15.1 mm vs. 17.1 mm, *p* = 0.038) or RVFWLS (-15.0% vs. -17.9%, *p* = 0.043). RV-PA uncoupling parameters such as TAPSE/sPAP, RVFWLS/sPAP and RV4CLS/sPAP were significantly lower in patients with primary endpoint, compared with those without (0.38 vs. 0.50 mm/mmHg, *p* = 0.001; 0.39 vs. 0.49%/mmHg, *p* = 0.001; 0.28 vs. 0.39%/mmHg, *p* = 0.006).

When considering only NAC Ia patients (*n* = 18), primary endpoint occurred in 6 patients (33%) (Table 2). RV-PA uncoupling parameters such TAPSE/sPAP, RVFWLS/sPAP and RV4CLS/sPAP were significantly lower in patients with primary endpoint, compared with those without (0.41 vs. 0.78 mm/mmHg, *p* = 0.013; 0.47 vs. 0.79 -%/mmHg, *p* = 0.042; 0.32 vs. 0.61 -%/mmHg, *p* = 0.041, respectively).

**Table 2:** RV-PA coupling characteristics according to endpoint occurrence in patients with wtATTR-CM stage NAC Ia

	No endpoint *N* = 12	Endpoint *N* = 6	*p*
TAPSE/sPAP (mm/mmHg)	0.78 (0.60–0.90)	0.41 (0.37–0.45)	**0.013**
RVFWLS/sPAP (%/mmHg)	0.79 (0.61–0.96)	0.47 (0.32–0.61)	**0.042**
RV4CLS/sPAP (%/mmHg)	0.61 (0.43–0.82)	0.32 (0.20–0.44)	**0.041**

Abbreviations as in Table 1

## Prognostic value of RV-PA uncoupling

**Fig. 2:**
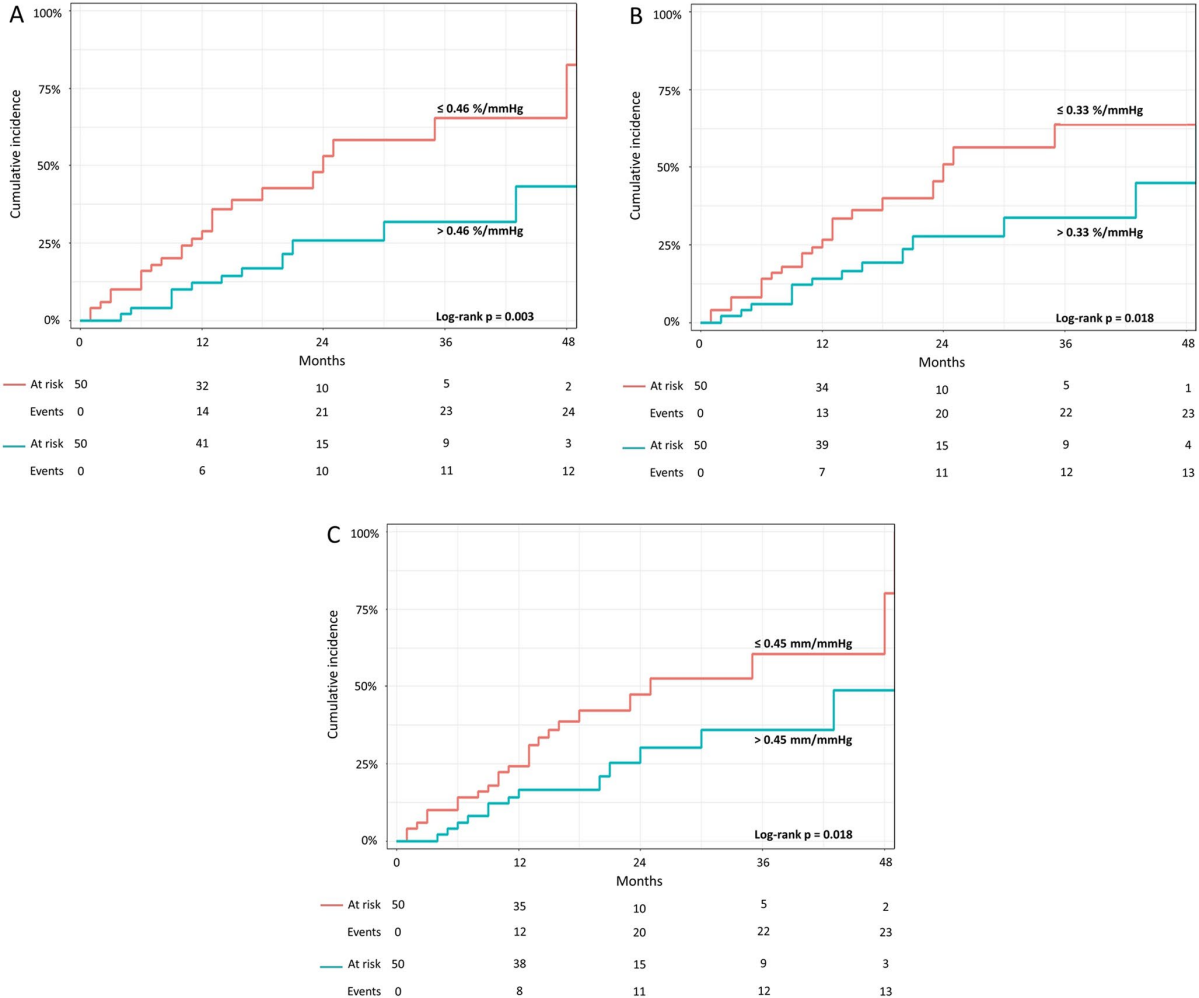
Cumulative incidence of composite endpoint according to the presence of RVFWSL/sPAP ≤ 0.46%/mmHg (**A**), RV4CLS/sPAP ≤ 0.33%/mmHg (**B**) or TAPSE/sPAP ≤ 0.45 mm/mmHg (**C**), showing bad outcome in patients with worse RV-PA coupling ratios. Abbreviations: RV4CLS = right ventricular four-chamber strain (including the septum); RVFWLS = right ventricular free-wall longitudinal strain; sPAP = systolic pulmonary artery pressure; TAPSE = tricuspid annulus plane systolic excursion

**Fig. 3:**
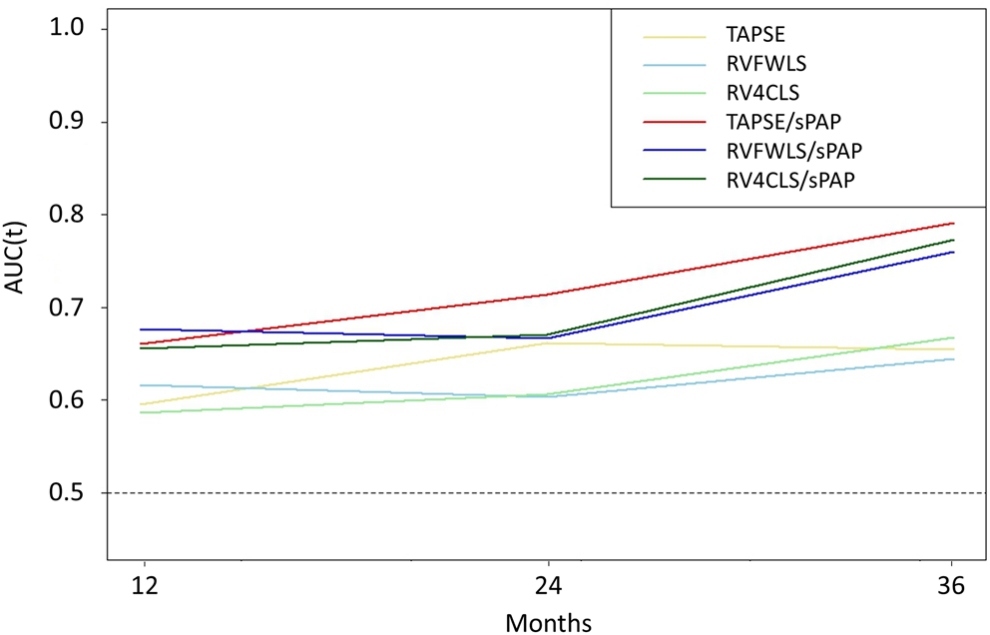
Time dependent AUC analysis, showing significant differences in prognostic value among RV systolic function indexes and RV-PA coupling ratios. Abbreviations as Fig. 2

Univariable analyses and all derived multivariable models are presented in Tables 3 and 4 RV-PA uncoupling emerged as independent predictor of composite endpoint, evaluated with TAPSE/sPAP (HR 0.04, 95% CI 0.01–0.24, *p* < 0.001), RVFWLS/sPAP (HR 0.07, 95% CI 0.01–0.41, *p* = 0.003) or RV4CLS/sPAP (HR 0.06, 95% CI 0.01–0.53, *p* = 0.011) ratios, and were confirmed after proportional hazards assumption analysis (Supplemental Table 2). Using median values for discriminating the composite endpoint (Fig. 2), 12-months cumulative incidence was significantly higher in patients with TAPSE/sPAP ≤ 0.45 mm/mmHg (24% vs. 16%, log–rank p 0.018), RVFWLS/sPAP ≤ 0.46%/mmHg (29% vs. 12%, log-rank *p* = 0.003) or RV4CLS/sPAP ≤ 0.33%/mmHg (27% vs. 14%, log-rank *p* = 0.018), respectively. To further investigate and compare the predictive value of RV-PA uncoupling data with RV function parameters alone, a time-dependent ROC curve analysis was performed. At 36 months, time dependent AUC for TAPSE, RVFWLS and RV4CLS were 0.65, 0.64 and 0.66, respectively. On the other hand, time dependent AUC for RVFWLS/sPAP, RV4CLS/sPAP, TAPSE/sPAP were 0.76, 0.77 and 0.79, respectively, without significantly differences between them (all p values > 0.05, Fig. 3). Nevertheless, these values were significantly higher than that of RV function parameters considered alone, resulting in a further incremental prognostic accuracy for the composite endpoint (all adjusted p values < 0.05). The same held true when compared with diastolic function parameters and daily high furosemide doses (Supplemental Fig. 2).

**Table 3:** Univariable analysis for composite endpoint predictors at 60-months follow up according to Cox’s regression

	Univariate analysis	
	HR (95% CI)	*p*
Age	1 (0.95–1.05)	0.9
Sex	0.64 (0.15–2.66)	0.5
HF presentation	2.73 (1.31–5.69)	**0.007**
LQRSV	0.59 (0.27–1.29)	0.2
QRS duration	1.02 (1.00–1.03)	**0.012**
NTproBNP	1.00 (1.00–1.00)	**0.004**
eGFR	0.99 (0.97–1)	0.2
TnI	1.01 (1.00–1.01)	0.1
Furosemide > 50 mg	2.87 (1.46–5.62)	**0.002**
Disease modifying therapy*	0.48 (0.22–1.07)	0.07
RWT	0.27 (0.05–1.56)	0.1
LV EF	0.98 (0.95–1.01)	0.3
LV Svi	1.00 (0.96–1.04)	0.8
LV GLS	1.07 (0.98–1.16)	0.1
E/A	1.77 (1.09–2.87)	**0.020**
E/e’	1.02 (0.97–1.07)	0.4
E/e’ > 14	3.19 (1.24–8.23)	**0.016**
Restrictive filling pattern	1.74 (0.90–3.36)	0.1
RALS	0.92 (0.83–1.00)	**0.048**
RV EDAi	1.16 (1.05–1.29)	**0.005**
TAPSE	0.92 (0.86–0.99)	**0.032**
FAC	0.98 (0.94–1.02)	0.3
RVFWLS	0.94 (0.89–0.99)	**0.022**
RV4CLS	0.93 (0.86–1.00)	**0.043**
sPAP	1.04 (1.02–1.07)	**0.001**
TAPSE/sPAP	0.004 (0.01–0.21)	**< 0.001**
RVFWLS/sPAP	0.12 (0.03–0.52)	**0.005**
RV4CLS/sPAP	0.06 (0.01–0.43)	**0.005**

Abbreviations as in Table 1 plus CI = confidence interval; HR = hazard ratio. *Time dependent Cox’s regression

**Table 4:** Multivariable models for prediction of composite endpoint at 60 months follow up according to Cox’s regression

	Model 1		Model 2		Model 3		Model 4		Model 5		Model 6	
	HR (95% CI)	*p*	HR (95% CI)	*p*	HR (95% CI)	*p*	HR (95% CI)	*p*	HR (95% CI)	*p*	HR (95% CI)	*p*
HF presentation	1.39 (0.61–3.18)	0.4	1.34 (0.57–3.16)	0.5	1.36 (0.56–3.28)	0.5	1.30 (0.59–2.85)	0.5	1.12 (0.50–2.55)	0.8	1.12 (0.49–2.57)	0.8
NTproBNP	1.00 (1.00–1.00)	0.5	1.00 (1.00–1.00)	0.2	1.00 (1.00–1.00)	0.2	1.00 (1.00–1.00)	0.7	1.00 (1.00–1.00)	0.7	1.00 (1.00–1.00)	0.5
Furosemide > 50 mg	2.62 (1.23–5.62)	**0.013**	2.42 (1.13–5.19)	**0.023**	2.47 (1.15–5.27)	**0.020**	3.38 (1.55–7.35)	**0.002**	2.77 (1.28–5.96)	**0.009**	2.74 (1.27–5.88)	**0.010**
TAPSE	0.94 (0.86–1.02)	0.2										
RVFWLS			0.97 (0.91–1.03)	0.3								
RV4CLS					0.97 (0.89–1.06)	0.5						
TAPSE/sPAP							0.04 (0.01–0.24)	**< 0.001**				
RVFWLS/sPAP									0.07 (0.01–0.41)	**0.003**		
RV4CLS/sPAP											0.06 (0.01–0.53)	**0.011**
Model C-index	0.71		0.69		0.69		0.74		0.73		0.72	
CV model C-index	0.69		0.65		0.68		0.73		0.70		0.70	

Abbreviations as in Table 2 plus CV = cross-validated

## Discussion

This study was designed to investigate the prognostic value of RV-PA uncoupling in a modern cohort of patients with wtATTR-CM. The main results were the following: (i) RV-PA uncoupling, evaluated with either RVFWLS/sPAP, RV4CLS/sPAP, or TAPSE/sPAP, was independently associated with the risk of the composite outcome of all-cause death or HF hospitalisation in patients with wtATTR-CM; (ii) in the earliest stage of disease, RV-PA uncoupling remained associated with poor outcome; (iii) RV-PA uncoupling indexes, such as RVFWLS/sPAP, RV4CLS/sPAP, or TAPSE/sPAP, showed incremental value in outcome prediction over TAPSE, RV4CLS, RVFWLS and sPAP, considered as separate parameters.

The recent development of non-invasive algorithms for the diagnosis of ATTR-CM, together with the rapidly evolving therapeutic landscape, has transformed wtATTR-CM from a rare and untreatable condition to a more prevalent disease, now diagnosed at earlier and milder stages. As was observed in the ATTRibute-CM [34] and HELIOS-B trial [35], patients are now less likely to have cardiovascular events than in the ATTR-ACT trial era [36], so that it is of great importance to identify early predictors of outcome in modern cohorts of wtATTR-CM patients, that can help risk stratify them and guide the prompt initiation of disease-modifying therapy.

The use of non–invasive surrogates of RV-PA uncoupling for prognosis prediction is not novel in the literature. Guazzi et al. investigated the independent prognostic significance of TAPSE/sPAP in 387 patients with different HFpEF aetiologies and found that TAPSE/sPAP < 0.35 mm/mmHg was independently associated with the risk of combined endpoint of HF hospitalisation or all-cause death [37]. Same results were demonstrated in our cohort, although with a higher TAPSE/sPAP cut-off value (0.45 mm/mmHg). Discrepancy could be due to lower sPAP data in our population, far less characterized by patients with chronic pulmonary diseases.

RV-PA uncoupling can be assessed also by means of RV strain function indexes, such as RVFWLS and RV4CLS [10–13], which are as known less angle- and volume dependent than TAPSE, and have a higher sensitivity for detecting subclinical RV systolic dysfunction. Bosch et al. investigated the contribution of RV dysfunction in 219 patients with HFpEF and found that RVFWLS/sPAP was independently associated with the risk of composite endpoint of all-cause mortality and HF hospitalisation [15]. This was confirmed by our study results, although with some difference in RVFWLS/sPAP cut-off value (lower in our cohort), possibly due to different pathophysiology of RV dysfunction in ATTR-CM compared to other HFpEF aetiologies. Indeed, other than pulmonary hypertension secondary to LV disease, in ATTR-CM there might be a direct contribution in RV systolic impairment also caused by myocardial amyloid deposition [38]. This pathophysiological mechanism is also suggested by the only moderate correlation between diastolic function and RV-PA parameters in our cohort.

The prognostic role of RV-PA uncoupling has been recently studied in patients with CA. In a mixed AL-CA and ATTR-CM cohort, Tomasoni et al. showed that the TAPSE/PASP ratio (median value 0.45 mm/mmHg) is a strong and independent predictor of all-cause death or HF hospitalisation, providing incremental risk prediction beyond TAPSE or sPAP considered alone [7]. A subsequent study confirmed these findings, although in a smaller mixed AL-CA and ATTR-CM cohort [8]. Our study results refined and expanded the prognostic role of RV-PA uncoupling in a modern cohort of ATTR-CM. To the best of our knowledge, this is the first study to focus on wtATTR-CM only and to apply and investigate multiple indexes of RV-PA uncoupling in this setting. Compared with the above-mentioned studies [7, 8], our wtATTR-CM patients were mostly characterised by earlier and milder disease stages (85% in NAC stage *≤* 2, 18% in NAC stage Ia and 82% in NYHA class *≤* II). Nonetheless, RV-PA uncoupling, either using M-mode or strain-based RV systolic function parameters, remained an independent predictor of poor outcome, thus suggesting that the maladaptation of RV-PA afterload could be a relatively early phaenomenon in the natural history of ATTR-CM, possibly driven by initial amyloid deposition in the basal segments of RV [39, 40]. The early and major involvement of basal regions of RV could also explain the non-incremental prognostic accuracy for poor prognosis of speckle-tracking based uncoupling indexes compared to M-mode one (Fig. 1, panel B). The different regional amyloid deposition in RV could also account for the overall reduced RALS in our cohort.

According to our study results, the evaluation of RV-PA uncoupling may aid in individual risk assessment and treatment selection. The higher risk of HF hospitalisation and mortality in asymptomatic, early stage (NAC Ia) patients with RV-PA uncoupling signs would support the need of immediate and more aggressive therapy, including not only disease-modifying, but also conventional HF drugs. Since wtATTR-CM mostly affects the older population [5] and encompasses a wide spectrum of disease severity, a more comprehensive staging system beyond NYHA class and classical biomarkers is needed to improve prognostic precision and optimize treatment strategies for individual patients.

## Limitations

This study has some limitations. First, the design is observational and single-centre, with a relatively small sample size (albeit in line with previous studies on the same topic [41, 44]), that may limit statistical power and generalizability of the findings. Nevertheless, methodological approach and data analysis depth are to be considered, together with the peculiar characteristics of our cohort, including mostly early diagnosed and less sick wtATTR-CM patients, thus reflecting more faithfully the current real– world population of patients [5]. Second, despite the availability of data about disease-modifying therapy, the evaluation of its efficacy could be affected by the small sample size and different timing of prescription between patients during follow up. Nevertheless, the most appropriate data analysis was employed, and the emerged early prognostic value of the RV-PA uncoupling assessment could highlight the necessity of further studies specifically addressing this important topic.

## Conclusions

In a modern cohort of patients with wtATTR-CM, RV-PA uncoupling emerged as an early and strong predictor of outcome, being independently associated with the risk of HF hospitalisation or all– cause death. No differences in risk prediction were observed among M-mode and strain-based RV function parameters. The evaluation of RV-PA uncoupling should be considered in the clinical practice for risk stratification and prognosis assessment of patients with wtATTR-CM, with potential implications treatment strategies definition.

## Electronic supplementary material

Below is the link to the electronic supplementary material.

Supplementary Material 1

## Data Availability

The data underlying this article will be shared on reasonable request to the corresponding author.

## References

[1] Wechalekar AD, Gillmore JD, Hawkins PN (2016) Systemic amyloidosis. Lancet 387:2641–2654 26719234 10.1016/S0140-6736(15)01274-X

[2] Fontana M, Ćorović A, Scully P et al (2019) Myocardial amyloidosis: the exemplar interstitial disease. JACC Cardiovasc Imaging 12:2345–2356 31422120 10.1016/j.jcmg.2019.06.023

[3] Quarta CC, Kruger JL, Falk RH (2012) Cardiac amyloidosis. Circulation 126:e178–e182 22988049 10.1161/CIRCULATIONAHA.111.069195

[4] Gillmore JD, Maurer MS, Falk RH et al (2016) Nonbiopsy diagnosis of cardiac transthyretin amyloidosis. Circulation 133:2404–2412 27143678 10.1161/CIRCULATIONAHA.116.021612

[5] Ioannou A, Patel RK, Razvi Y et al (2022) Impact of earlier diagnosis in cardiac ATTR amyloidosis over the course of 20 years. Circulation 146:1657–1670 36325894 10.1161/CIRCULATIONAHA.122.060852 PMC9698091

[6] Aimo A, Castiglione V, Rapezzi C et al (2022) RNA-targeting and gene editing therapies for transthyretin amyloidosis. Nat Rev Cardiol 19:655–667 35322226 10.1038/s41569-022-00683-z

[7] Tomasoni D, Adamo M, Porcari A et al (2023) Right ventricular to pulmonary artery coupling and outcome in patients with cardiac amyloidosis. Eur Heart J Cardiovasc Imaging 24:1405–1414 37379445 10.1093/ehjci/jead145

[8] Palmiero G, Monda E, Verrillo F et al (2023) Prevalence and clinical significance of right ventricular pulmonary arterial uncoupling in cardiac amyloidosis. Int J Cardiol 388:131147 37423570 10.1016/j.ijcard.2023.131147

[9] Pestelli G, Fiorencis A, Trevisan F et al (2021) New measures of right ventricle-pulmonary artery coupling in heart failure: an all-cause mortality echocardiographic study. Int J Cardiol 329:234–241 33359279 10.1016/j.ijcard.2020.12.057

[10] Richter MJ, Rako ZA, Tello K (2023) Ratio between right ventricular strain and systolic pulmonary artery pressure as a surrogate for right ventricular to pulmonary arterial coupling: validation against the gold standard. Eur Heart J Cardiovasc Imaging 24:e50–e52 36546641 10.1093/ehjci/jeac253

[11] Brener MI, Grayburn P, Lindenfeld J et al (2021) Right Ventricular-Pulmonary arterial coupling in patients with HF secondary MR: analysis from the COAPT trial. JACC Cardiovasc Interv 14:2231–2242 34674862 10.1016/j.jcin.2021.07.047

[12] Iacoviello M, Monitillo F, Citarelli G et al (2017) Right ventriculo-arterial coupling assessed by two-dimensional strain: A new parameter of right ventricular function independently associated with prognosis in chronic heart failure patients. Int J Cardiol 241:318–321 28479093 10.1016/j.ijcard.2017.04.051

[13] Ünlü S, Bézy S, Cvijic M et al (2023) Right ventricular strain related to pulmonary artery pressure predicts clinical outcome in patients with pulmonary arterial hypertension. Eur Heart J Cardiovasc Imaging 24:635–642 35852912 10.1093/ehjci/jeac136

[14] Guazzi M, Bandera F, Pelissero G et al (2013) Tricuspid annular plane systolic excursion and pulmonary arterial systolic pressure relationship in heart failure: an index of right ventricular contractile function and prognosis. Am J Physiol Heart Circ Physiol 305:H1373–H1381 23997100 10.1152/ajpheart.00157.2013

[15] Bosch L, Lam CSP, Gong L et al (2017) Right ventricular dysfunction in left-sided heart failure with preserved versus reduced ejection fraction. Eur J Heart Fail 19:1664–1671 28597497 10.1002/ejhf.873

[16] Lyhne MD, Kabrhel C, Giordano N et al (2021) The echocardiographic ratio tricuspid annular plane systolic excursion/pulmonary arterial systolic pressure predicts short-term adverse outcomes in acute pulmonary embolism. Eur Heart J Cardiovasc Imaging 22:285–294 33026070 10.1093/ehjci/jeaa243

[17] Trousselle L, Eggenspieler F, Huttin O, Pace N, Nazeyrollas P, Faroux L et al (2024) Echocardiographic assessment of right ventricular function and right ventriculoarterial coupling in tricuspid regurgitation. Int J Cardiovasc Imaging 40(11):2247–2259 39225749 10.1007/s10554-024-03215-7

[18] Roccabruna A, Fortuni F, Comuzzi A, Armani I, Bolzan B, Franchi E et al (2024) Right ventricular-pulmonary artery coupling in patients undergoing cardiac resynchronization therapy. Int J Cardiovasc Imaging 40(11):2325–2334 39235726 10.1007/s10554-024-03233-5

[19] Mendes LF, Brandão M, Diaz SO, Almeida MC, Barros AS, Saraiva F et al (2024) Impact of right ventricle-pulmonary artery coupling in patients undergoing transcatheter aortic valve implantation. Int J Cardiovasc Imaging 40(8):1745–1753 38940965 10.1007/s10554-024-03165-0 PMC11401781

[20] Lillo R, Graziani F, Ingrasciotta G, Przbybylek B, Iannaccone G, Locorotondo G et al (2022) Right ventricle systolic function and right ventricle-pulmonary artery coupling in patients with severe aortic stenosis and the early impact of TAVI. Int J Cardiovasc Imaging 38(8):1761–1770 35230568 10.1007/s10554-022-02569-0

[21] Russo M, Obici L, Bartolomei I et al (2020) ATTRv amyloidosis Italian registry: clinical and epidemiological data. Amyloid 27:259–265 32696671 10.1080/13506129.2020.1794807

[22] Rapezzi C, Quarta CC, Obici L et al (2013) Disease profile and differential diagnosis of hereditary transthyretin-related amyloidosis with exclusively cardiac phenotype: an Italian perspective. Eur Heart J 34:520–528 22745357 10.1093/eurheartj/ehs123

[23] Quarta CC, Solomon SD, Uraizee I et al (2014) Left ventricular structure and function in transthyretin-related versus light-chain cardiac amyloidosis. Circulation 129:1840–1849 24563469 10.1161/CIRCULATIONAHA.113.006242

[24] Gillmore JD, Damy T, Fontana M et al (2018) A new staging system for cardiac transthyretin amyloidosis. Eur Heart J 39:2799–2806 29048471 10.1093/eurheartj/ehx589

[25] Law S, Bezard M, Petrie A et al (2022) Characteristics and natural history of early-stage cardiac transthyretin amyloidosis. Eur Heart J 43:2622–2632 35608040 10.1093/eurheartj/ehac259 PMC9279112

[26] Italian Agency of Pharmacy determina n. 15, published in G.U. 250/2021

[27] Lang RM, Badano LP, Mor-Avi V et al (2015) Recommendations for cardiac chamber quantification by echocardiography in adults: an update from the American society of echocardiography and the European association of cardiovascular imaging. J Am Soc Echocardiogr 28:1–39e14 25559473 10.1016/j.echo.2014.10.003

[28] Badano LP, Kolias TJ, Muraru D et al (2018) Standardization of left atrial, right ventricular, and right atrial deformation imaging using two-dimensional speckle tracking echocardiography: a consensus document of the EACVI/ASE/Industry task force to standardize deformation imaging. Eur Heart J Cardiovasc Imaging 19:591–600 29596561 10.1093/ehjci/jey042

[29] Badano LP, Muraru D, Parati G et al (2020) How to do right ventricular strain. Eur Heart J Cardiovasc Imaging 21:825–827 32504092 10.1093/ehjci/jeaa126

[30] Rudski LG, Lai WW, Afilalo J et al (2010) Guidelines for the echocardiographic assessment of the right heart in adults: a report from the American society of echocardiography endorsed by the European association of echocardiography, a registered branch of the European society of cardiology, and the Canadian society of echocardiography. J Am Soc Echocardiogr 23:685–713 20620859 10.1016/j.echo.2010.05.010

[31] Cheng RK, Levy WC, Vasbinder A et al (2020) Diuretic dose and NYHA functional class are independent predictors of mortality in patients with transthyretin cardiac amyloidosis. JACC CardioOncol 2:414–424 33073249 10.1016/j.jaccao.2020.06.007 PMC7561022

[32] Tini G, Milani P, Zampieri M et al (2023) Diagnostic pathways to wild-type transthyretin amyloid cardiomyopathy: a multicentre network study. Eur J Heart Fail 25:845–853 36907828 10.1002/ejhf.2823

[33] Blanche P, Dartigues JF, Jacqmin-Gadda H (2013) Estimating and comparing time-dependent areas under receiver operating characteristic curves for censored event times with competing risks. Stat Med 32:5381–5397 24027076 10.1002/sim.5958

[34] Gillmore JD, Judge DP, Cappelli F et al (2024) Efficacy and safety of acoramidis in transthyretin amyloid cardiomyopathy. N Engl J Med 390(2):132–142 38197816 10.1056/NEJMoa2305434

[35] Fontana M, Berk JL, Gillmore JD, Witteles RM, Grogan M, Drachman B et al (2024) Vutrisiran in patients with transthyretin amyloidosis with cardiomyopathy. N Engl J Med 10.1056/NEJMc2501792 40334172

[36] Maurer MS, Schwartz JH, Gundapaneni B et al (2018) Tafamidis treatment for patients with transthyretin amyloid cardiomyopathy. N Engl J Med 379:1007–1016 30145929 10.1056/NEJMoa1805689

[37] Guazzi M, Dixon D, Labate V et al (2017) RV contractile function and its coupling to pulmonary circulation in heart failure with preserved ejection fraction: stratification of clinical phenotypes and outcomes. JACC Cardiovasc Imaging 10:1211–1221 28412423 10.1016/j.jcmg.2016.12.024

[38] Knight DS, Zumbo G, Barcella W et al (2019) Cardiac structural and functional consequences of amyloid deposition by cardiac magnetic resonance and echocardiography and their prognostic roles. JACC Cardiovasc Imaging 12:823–833 29680336 10.1016/j.jcmg.2018.02.016

[39] De Gaspari M, Sinigiani G, De Michieli L et al (2023) Relative apical sparing in cardiac amyloidosis is not always explained by an amyloid gradient. Eur Heart J Cardiovasc Imaging 24:1258–1268 37191052 10.1093/ehjci/jead107 PMC10445246

[40] Porcari A, Fontana M, Canepa M et al (2024) Clinical and prognostic implications of right ventricular uptake on bone scintigraphy in transthyretin amyloid cardiomyopathy. Circulation 149:1157–1168 38328945 10.1161/CIRCULATIONAHA.123.066524 PMC11000629

[41] Ozbay B, Satyavolu BS, Rearick C et al (2024) Right ventricular strain improves the echocardiographic diagnosis and risk stratification of transthyretin cardiac amyloidosis among other phenotypes of left ventricular hypertrophy. J Am Soc Echocardiogr 26:S0894-7317(24)00319-5 10.1016/j.echo.2024.06.006 38942217

[42] Moñivas Palomero V, Durante-Lopez A, Sanabria MT et al (2019) Role of right ventricular strain measured by Two-Dimensional echocardiography in the diagnosis of cardiac amyloidosis. J Am Soc Echocardiogr 32:845–853e1 31078369 10.1016/j.echo.2019.03.005

[43] Pradel S, Magne J, Jaccard A et al (2019) Left ventricular assessment in patients with systemic light chain amyloidosis: a 3-dimensional speckle tracking transthoracic echocardiographic study. Int J Cardiovasc Imaging 35:845–854 30623354 10.1007/s10554-018-01524-2

[44] Licordari R, Minutoli F, Recupero A et al (2021) Early impairment of right ventricular morphology and function in Transthyretin-Related cardiac amyloidosis. J Cardiovasc Echogr 31:17–22 34221881 10.4103/jcecho.jcecho_112_20 PMC8230159

