# Supplementary material for: Right ventricular to pulmonary artery uncoupling is an early predictor of poor outcome in wild-type transthyretin amyloid cardiomyopathy
Source: Int J Cardiovasc Imaging. 2025 Apr 10;41(6):1119–30. doi: 10.1007/s10554-025-03394-x (PMC12162735; doi:10.1007/s10554-025-03394-x)
Supplement: Supplementary file 1 — Supplementary Material 1 [file 10554_2025_3394_MOESM1_ESM.docx]

**Supplementary Methods**

**Clinical History and examination**

Careful clinical history was collected, including information about history of hypertension, diabetes, coronary artery disease, chronic obstructive pulmonary disease, pulmonary artery hypertension, atrial fibrillation, episodes of HF and pulmonary embolism of any aetiology. Height, weight, systemic blood pressure, ongoing medical therapy, including ATTR disease-modifying drugs, and data regarding New York Heart Association (NYHA) class and National Amyloidosis Centre (NAC) stage at baseline, including NAC Ia stage, were also collected. Body surface area was calculated with the Dubois formula.

**Electrocardiography**

12-lead ECGs were acquired in supine position with the limbs leads placed at the wrists and ankles at standard speed (25 mm/sec) and amplification (10 mm/mV) and retrospectively reviewed for rhythm and P wave, PQ interval and QRS complex characteristics and durations. Low QRS voltages were defined as QRS amplitude <5 mm (0.5 mV) in all peripheral leads, including both negative and positive components.

**Biomarkers**

N terminal pro-brain natriuretic peptide (NT-proBNP), high sensitivity troponin I and creatinine were also collected. Estimated glomerular filtration rate (eGFR) was calculated using the Cockroft-Gault formula.

**Echocardiography**

Inadequate image quality was defined in the presence of a frame rate < 50 frames per seconds or inability to accurately visualize the RV from base to apex or to perform adequate speckle - tracking analysis on any segments. Right ventricle free wall (RVFW) thickness was measured on 2D images in subcostal view in diastole, whereas chamber quantification, function and peak systolic longitudinal strain (LS) were assessed in RV-focused apical four chamber view. RV diastolic area was contoured in the frame immediately prior to tricuspid valve closure, whereas systolic area was contoured in the frame immediately prior the tricuspid valve opening. From these contours, fractional area change (FAC) was calculated. The definition of the beginning and the end of the RV ejection time was assessed by the pulsed-wave Doppler tracing recorded in the RV outflow tract. Right atrial longitudinal strain (RALS) was calculated using the optimized RV-focused apical four chamber view. The tracing started at the tricuspid valve annulus, included RA lateral wall, RA roof, RA septal wall, and ended at the opposite tricuspid annulus. End-diastole and onset of atrial contraction were defined according to the R- and P-waves in ECG as surrogates of tricuspid valve inflow profile. RA strain curve profile was used for checking the reliability of ECG-derived definitions.

**Supplemental Table 1 – population characteristics according to disease modifying therapy start during follow up.**

| **Variable** | **Total population**  **N = 100** | **No DMT**  **N = 47** | **DMT**  **N = 53** | **p** |
| --- | --- | --- | --- | --- |
| **Clinical characteristics** | | | | |
| Age (years) | 81 (75 – 85) | 85 (80 – 86) | 79 (74 – 81) | <0.001 |
| HF presentation (%) | 52 (54) | 28 (60) | 24 (45) | 0.2 |
| COPD (%) | 9 (9) | 2 (4) | 6 (12) | 0.2 |
| OSAS (%) | 1 (1) | 0 (0) | 1 (2) | 0.4 |
| NAC stage III (%) | 15 (15) | 9 (24) | 6 (12) | 0.1 |
| **Medical therapy** | | | | |
| Furosemide (%) | 69 (69) | 35 (75) | 34 (64) | 0.3 |
| Dose of furosemide (mg) | 25 (0 – 50) | 50 (0 – 75) | 25 (0 – 50) | 0.1 |
| β – blockers (%) | 53 (53) | 23 (49) | 30 (57) | 0.4 |
| ACE-i/ARBs/ARNI (%) | 50 (50) | 22 (47) | 28 (53) | 0.7 |
| SGLT2i (%) | 5 (5) | 3 (6) | 2 (4) | 0.7 |
| MRA (%) | 39 (39) | 20 (43) | 19 (36) | 0.5 |
| **Electrocardiogram characteristics** | | | | |
| AF (%) | 49 (49) | 23 (49) | 26 (49) | 1.0 |
| LQRSV (%) | 31 (31) | 9 (20) | 22 (42) | 0.02 |
| QRS duration (ms) | 118 (101 – 144) | 118 (104 – 145) | 105 (96 – 132) | 0.03 |
| **Biochemical characteristics** | | | | |
| NTproBNP (ng/L) | 1777 (815 – 4896) | 2111 (1021 – 6170) | 1518 (726 – 4225) | 0.1 |
| eGFR (ml/min/m2) | 60 (50 – 77) | 58 (45 – 77) | 62 (53 – 77) | 0.2 |
| Hs-TnI (ng/L) | 78 (41 – 138) | 95 (62 – 224) | 55 (31 – 92) | 0.001 |
| **Echocardiogram characteristics** | | | | |
| IVS (mm) | 18 (16 – 20) | 18 (15 – 19) | 18 (16 – 21) | 0.4 |
| RWT | 0.72 (0.59 – 0.83) | 0.70 (0.60 – 0.79) | 0.75 (0.57 – 0.86) | 0.5 |
| LV mass (gr) | 307 (258 – 387) | 312 (267 – 387) | 284 (249 – 399) | 0.6 |
| LV EDVi (ml/m2) | 56 (46 – 67) | 55 (46 – 70) | 57 (46 – 67) | 0.7 |
| LV EF (%) | 52 (44 – 57) | 50 (40 – 56) | 54 (46 – 58) | 0.2 |
| LV SVi (ml/m2) | 27.2 (22.2 – 33.1) | 26.6 (21.3 – 31.8) | 28.1 (23.2 – 34.5) | 0.2 |
| LV GLS (-%) | 11 (8 – 13) | 11 (7 – 13) | 11 (8 – 13) | 0.5 |
| E/e’ | 16 (13 – 19) | 16 (14 – 19) | 15 (12 – 21) | 0.3 |
| LAVi (ml/m2) | 52.5 (42.3 – 65.4) | 51.4 (41.9 – 69.6) | 53.2 (42.4 – 61.8) | 0.6 |
| RAVi (ml/m2) | 45.5 (34.4 – 57.1) | 45.9 (34.4 – 59.5) | 44.9 (34.6 – 56.3) | 0.5 |
| RV thickness (mm) | 7.0 (4.2 – 9.0) | 7.0 (5.0 – 9.0) | 7.4 (4.0 – 9.0) | 0.9 |
| RV EDAi (cm2/m2) | 11.6 (9.8 – 12.9) | 11.9 (0.7 – 13.3) | 11.4 (9.9 – 12.6) | 0.3 |
| TAPSE (mm) | 16.5 (13 – 20) | 16 (13 – 19.5) | 17 (12.9 – 20.4) | 0.5 |
| RV FAC (%) | 35.5 (30.0 – 41.8) | 35 (30 – 42) | 36 (31 – 42) | 0.8 |
| RVFWLS (-%) | 16.5 (12 – 21.5) | 17 (11 – 22) | 16 (12 – 22) | 0.9 |
| RV4CLS (-%) | 12.1 (9.1 – 16.6) | 12.2 (8.0 – 16.9) | 11.9 (8.9 – 16.5) | 0.7 |
| sPAP (mmHg) | 35 (26.3 – 45) | 37 (27 – 46) | 34 (25 – 43) | 0.4 |
| TAPSE/sPAP (mm/mmHg) | 0.45 (0.33 – 0.72) | 0.45 (0.31 – 0.68) | 0.48 (0.34 – 0.82) | 0.4 |
| RVFWLS/sPAP (%/mmHg) | 0.46 (0.31 – 0.72) | 0.44 (0.31 – 0.61) | 0.48 (0.31 – 0.75) | 0.4 |
| RV4CLS/sPAP (%/mmHg) | 0.33 (0.23 – 0.52) | 0.31 (0.21 – 0.48) | 0.35 (0.23 – 0.59) | 0.4 |
| Trivial TR (%) | 34 (34) | 17 (36) | 17 (32) | 0.7 |
| Mild TR (%) | 45 (45) | 21 (45) | 24 (45) | 1.0 |
| Moderate TR (%) | 20 (20) | 9 (19) | 11 (21) | 1.0 |
| Severe TR (%) | 1 (1) | 0 (0) | 1 (2) | 1.0 |
| Severe MR (%) | 0 (0) | 0 (0) | 0 (0) | 1.0 |
| Severe pericardial effusion (%) | 1 (1) | 0 (0) | 1 (2) | 1.0 |
| Pleural effusion (%) | 8 (8) | 4 (9) | 4 (8) | 1.0 |
| **Endpoint** | | | | |
| Heart failure hospitalization (%) | 25 (25) | 17 (36) | 8 (15) | 0.02 |
| Death (%) | 22 (22) | 18 (38) | 4 (8) | <0.001 |

Quantitative variables expressed as median value (25^th^ – 75^th^ percentile). Qualitative variables expressed as absolute number (%). Abbreviations: ACE-i = Angiotensin converter enzyme inhibitor; AF = atrial fibrillation; ARBs = angiotensin receptor blockers; ARNI = angiotensin receptor neprilysin receptor inhibitors; COPD = chronic obstructive pulmonary disease; DMT = disease modifying therapy; EDAi = end diastolic area indexed; EDVi = end diastolic volume indexed; EF = ejection fraction; eGFR = estimated glomerular filtration rate; FAC = fractional area change; GLS = global longitudinal strain; HF = heart failure; Hs – TnI = high sensitivity troponin I; IVS = interventricular septum; LAVi = left atrium volume indexed; LQRSV = low QRS voltages; LV = left ventricle; MR = mitral regurgitation; MRA = mineralocorticoids receptor antagonist; NAC = National Amyloid Centre; NYHA = New York Heart Association; NTproBNP = N-Terminal pro brain natriuretic peptide; OSAS = obstructive sleep apnoea syndrome; PW = posterior wall; RAVi = right atrium volume indexed; RVFWLS = RV free wall longitudinal strain; RV4CLS = RV 4-chamber longitudinal strain; RWT = relative wall thickness; SGLT2i = Sodium glucose transporter 2 inhibitors; sPAP = systolic pulmonary artery pressure; SVi = stroke volume indexed; RV = right ventricle; TAPSE = tricuspid annulus plane systolic excursion; TR = tricuspid regurgitation.

**Supplemental Table 2.** Intraclass correlation coefficients (ICC) for intra- and inter-readers variability assessment.

|  | **Intrareader ICC** | **Interreaders ICC** |
| --- | --- | --- |
| **TAPSE/sPAP** | 0.96 (0.94 – 0.97) | 0.88 (0.83 – 0.92) |
| **RVFWLS/sPAP** | 0.97 (0.96 – 0.98) | 0.87 (0.82 – 0.91) |
| **RV4CLS/sPAP** | 0.93 (0.90 – 0.95) | 0.82 (0.74 – 0.88) |

**Supplemental Figure 1.** CONSORT flow diagram**.**

**
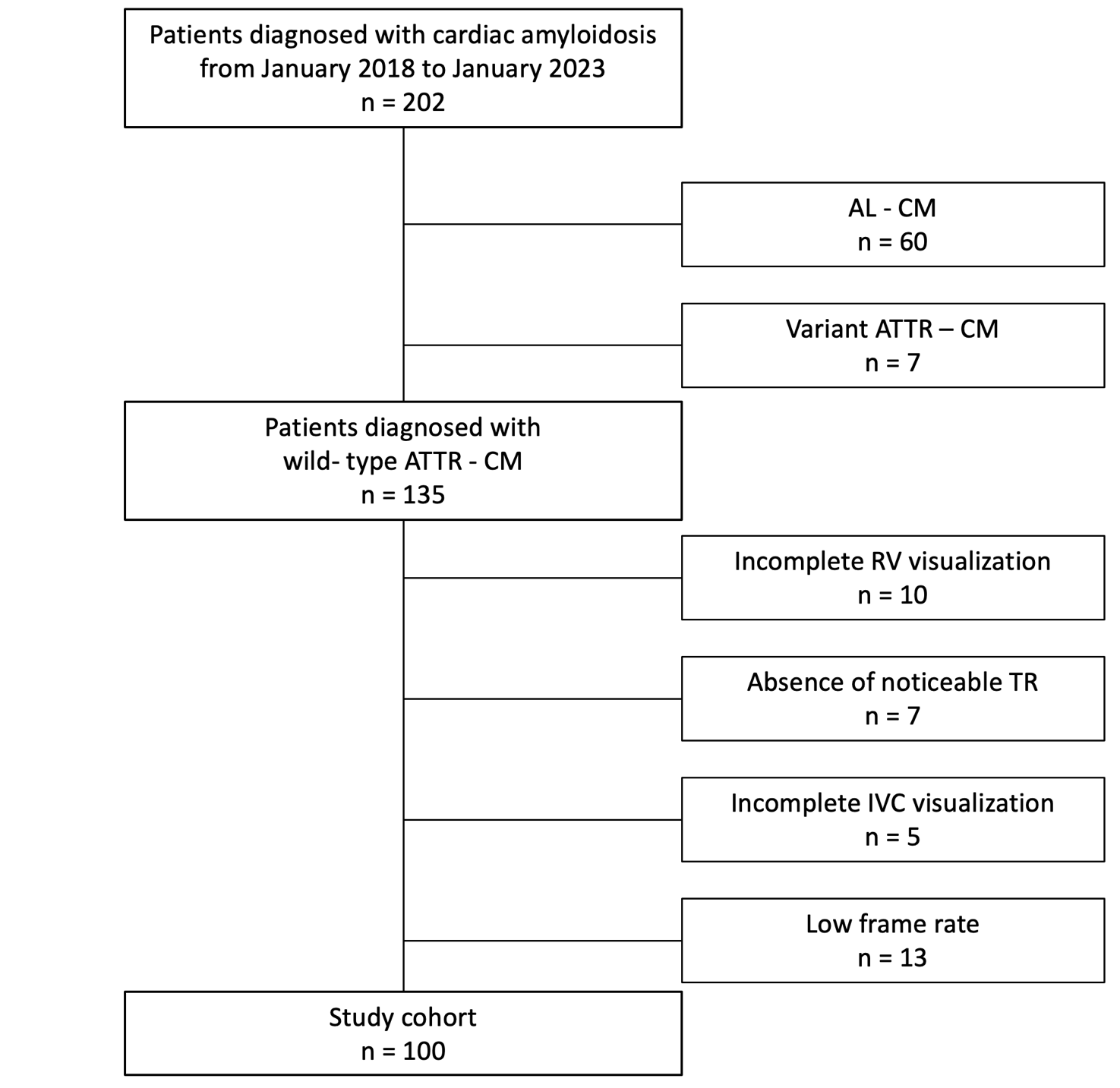
**

**Legend.** CONSORT flow diagram showing the flow of patients through the study.

AL-CM= light-chains amyloid cardiomyopathy; ATTR-CM= transthyretin amyloid cardiomyopathy; IVC= inferior vena cava; RV= right ventricle; TR=tricuspid regurgitation.

**Supplemental Figure 2.** Time dependent AUC analysis, showing significant differences in prognostic value among RV systolic function indexes and RV-PA coupling ratios.


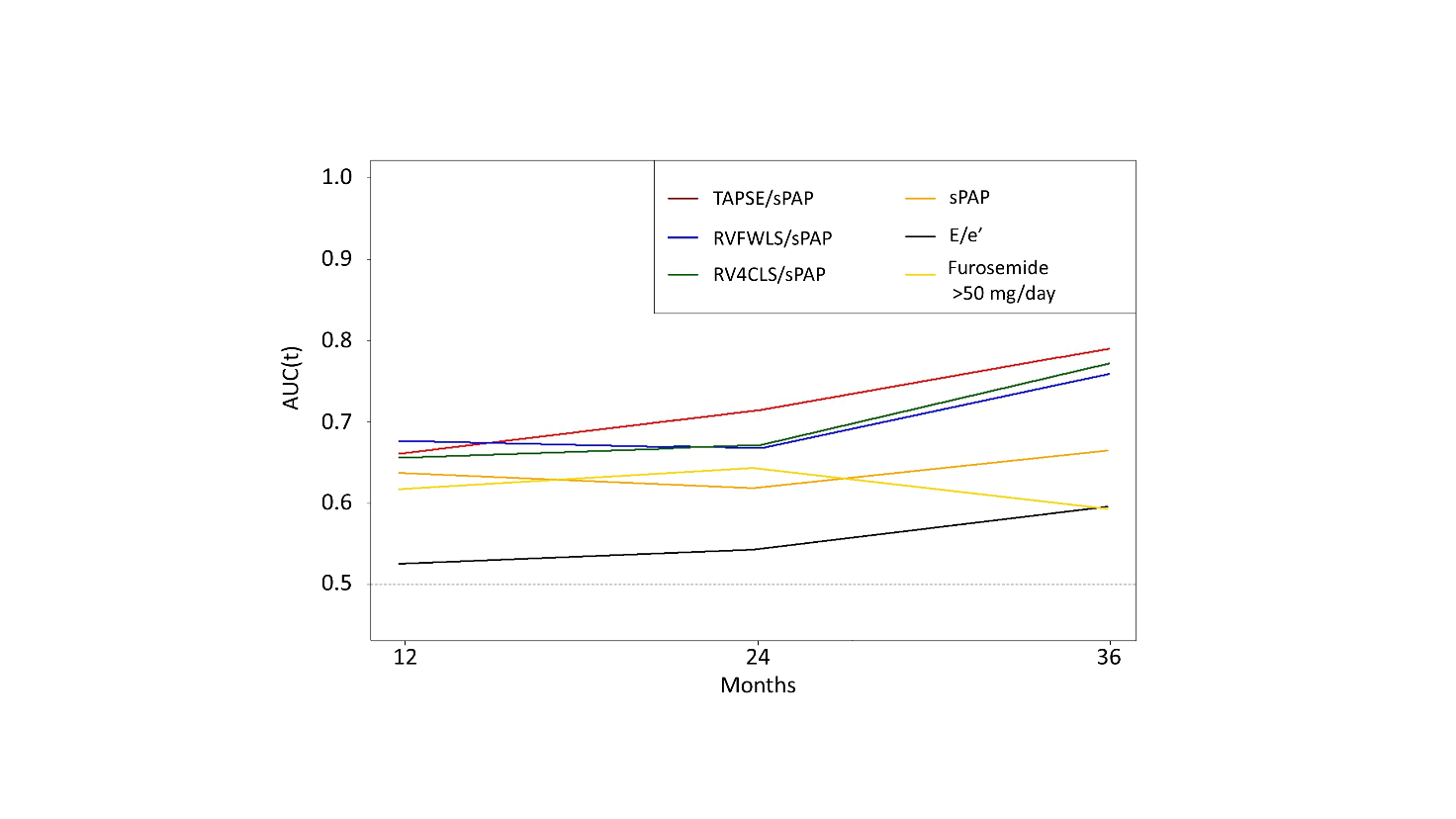


**Supplemental Figure 3.** Scatter plots to investigate relationship between E/e’ ratio and TAPSE/sPAP (Panel A), RVFWLS/sPAP (Panel B) and RV4CLS/sPAP (Panel C).

**
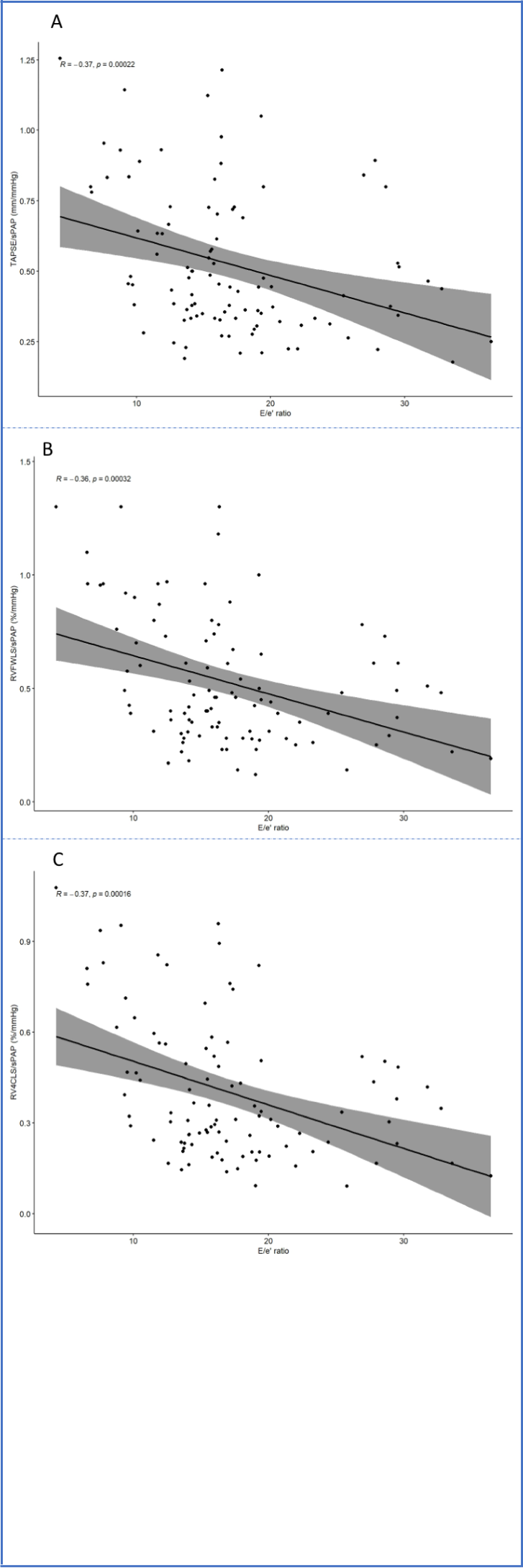
**
